# NAD^+^‒circadian rhythm coupling in dementia

**DOI:** 10.1002/alz.71360

**Published:** 2026-05-01

**Authors:** Shi‐qi Zhang, Jiyeon Lee, Jun‐ping Pan, Rune Enger, Harald Hrubos‐Strøm, Erik S. Musiek, Evandro Fei Fang, Weidong Le

**Affiliations:** ^1^ Key Laboratory of Liaoning Province for Research on the Pathogenic Mechanisms of Neurological Diseases The First Affiliated Hospital, Dalian Medical University Dalian China; ^2^ Department of Clinical Molecular Biology University of Oslo and Akershus University Hospital Lørenskog Norway; ^3^ Center for Convergence Research of Neurological Disorders Ajou University School of Medicine Suwon South Korea; ^4^ Letten Centre and GliaLab Division of Anatomy Department of Molecular Medicine Institute of Basic Medical Sciences University of Oslo Oslo Norway; ^5^ Department of Neurosurgery Oslo University Hospital, Rikshospitalet Oslo Norway; ^6^ K. G. Jebsen Centre for Brain Fluid Research Oslo Norway; ^7^ Institute of Clinical Medicine Faculty of Medicine University of Oslo Oslo Norway; ^8^ Division of Surgery Department of Otorhinolaryngology Akershus University Hospital Nordbyhagen Norway; ^9^ Department of Neurology and Center on Biological Rhythms and Sleep Washington University in St. Louis School of Medicine St. Louis Missouri USA; ^10^ Norwegian Centre on Healthy Ageing (NO‐Age) and Norwegian National Anti‐Alzheimer's Disease (NO‐AD) Network Oslo Norway; ^11^ Neurology Program Sir Run Run Shaw Hospital Zhejiang University School of Medicine Hangzhou China

**Keywords:** CD38, circadian rhythm, dementia, NAD+, PARP1, SIRT1, sleep

## Abstract

The circadian rhythm system and sleep coordinate whole‐body functions across the 24‐h cycle, yet these rhythms progressively deteriorate with neurodegenerative diseases, including dementia. Growing evidence indicates that nicotinamide adenine dinucleotide (NAD^+^) interacts with the circadian system through multiple molecular pathways and that NAD^+^ levels decline with dementia. In this review, we synthesize current evidence on the bidirectional relationship between NAD^+^ metabolism and circadian regulation in several dementia disorders, emphasizing the key circadian pathways, the nicotinamide phosphoribosyltransferase‐mediated salvage synthesis, the NAD^+^/sirtuins‐dependent signaling, and the consumption of NAD^+^ by PARP1 and CD38. Finally, we also examine pharmacological and lifestyle strategies that target NAD^+^, including NAD^+^ precursors, modulators of NAD^+^ biosynthetic and depleting enzymes, timed light and activity exposure, structured exercise programs, and dietary interventions. Overall, we focus on the bidirectional interplay between NAD^+^ metabolism and circadian rhythm regulation in dementia, with particular emphasis on how this interaction influences sleep and cognitive phenotypes across different dementia subtypes.

**Trial Registration**: ClinicalTrials.gov identifier: NCT05040321, NCT04430517, NCT06971224, NCT05500170, NCT04070378

## Highlights

- We synthesize evidence on the clinical features and mechanisms of circadian and sleep disturbances across dementia subtypes, including their potential links to NAD^+^.
- NAMPT, SIRT1, PARP1, and CD38 signaling mediate bidirectional coupling between NAD^+^ metabolism and the circadian clock.
- Key links between NAD^+^ signaling and TTFL components are summarized, with interactions requiring further confirmation highlighted.
- We synthesize evidence for NAD^+^‐driven mechanisms of circadian disruption in dementia for further exploration.
- Pharmacological and lifestyle‐based strategies targeting NAD^+^ restoration are outlined as potential approaches to improve sleep and circadian rhythm integrity.

## 1 INTRODUCTION

### 1.1 Circadian rhythm and sleep disorders in dementia

Sleep supports stable brain and body function by aligning physiology with the light‐dark cycle. Light entrains the master circadian pacemaker, located in the suprachiasmatic nucleus (SCN) of the hypothalamus, which in turn regulates autonomic output and drives nocturnal melatonin synthesis in the pineal gland.^1^, ^2^ The sleep‐wake cycle is regulated by a reciprocally inhibitory switch network linking the ventrolateral preoptic (VLPO) area of the hypothalamus with arousal‐promoting systems, reinforced by orexin neurons.^3^, ^4^ During sleep, the VLPO area, via primarily GABAergic and galaninergic neurons, suppresses multiple arousal centers. During wakefulness, orexin neurons in the lateral hypothalamus provide excitatory drive to arousal nuclei and counteract VLPO‐mediated inhibition, thereby consolidating wakefulness and limiting inappropriate state transitions.^5^, ^6^ In parallel, the SCN conveys circadian timing information through hypothalamic and thalamic relay nodes, including the subparaventricular zone and dorsomedial hypothalamus, thereby modulating VLPO excitability and shaping daily sleep propensity.^4^

Dementia usually displays neuronal function declines, weakening SCN output, diminishment of circadian amplitude, fragmented sleep, and blunted daily activity.^7^, ^8^, ^9^, ^10^, ^11^, ^12^ With dementia processing and aging, the diurnal oscillation amplitude of behavioral circadian rhythms declines, clock‐controlled gene expression in the brain is dysregulated, and levels of circadian rhythm‐related metabolites such as oxidized nicotinamide adenine dinucleotide (NAD^+^) also decrease.^13^, ^14^, ^15^, ^16^, ^17^, ^18^, ^19^, ^20^, ^21^, ^22^ These circadian‐associated alterations may disrupt sleep‐wake regulation and thereby contribute to cognitive decline, impaired executive function, and increased risk of neurodegenerative diseases. Accordingly, maintaining sleep continuity and following regular timing of light exposure, activity, and meals are core strategies for preserving cognitive resilience and delaying dementia.

### 1.2 Molecular clock architecture

**FIGURE 1:**
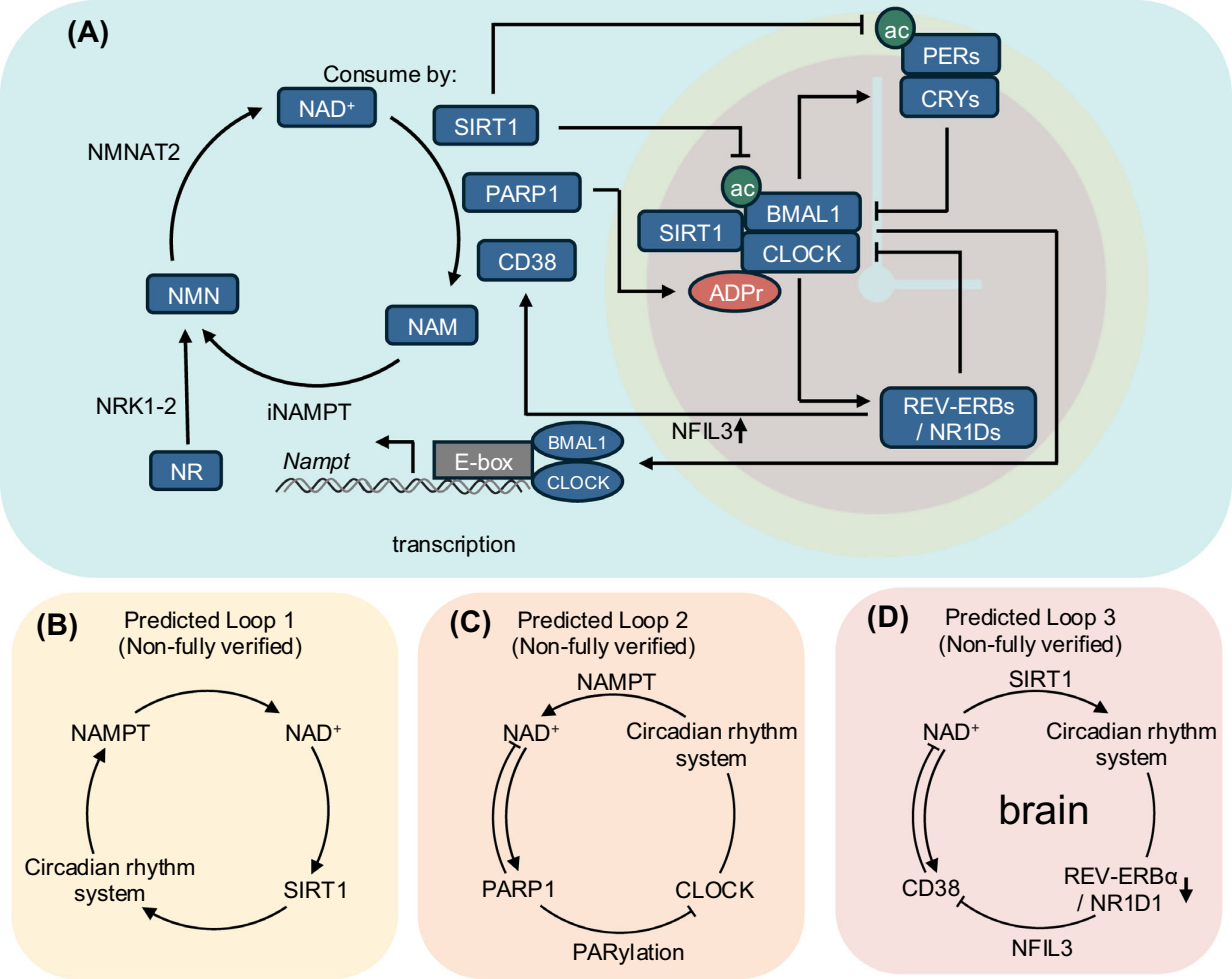
Bidirectional coupling between NAD^+^ metabolism and the circadian clock. (A) The panel illustrates the NAD^+^ salvage cycle, in which extracellular NR and NMN are converted through NR kinase 1/2 (NRK1/2) and NMNAT2, and intracellular *Nampt* regenerates NAD^+^ from NAM. NAD^+^ fuels SIRT1 as well as the NAD+‐consuming enzymes PARP1 and CD38. NAD^+^ activates SIRT1, which deacetylates BMAL1 and PERs complexes to regulate core clock transcription. PARP1 uses NAD^+^ to add ADP‐ribose chains to CLOCK, thereby modulating clock output. CD38 degrades NAD+ and is regulated by CLOCK‐controlled REV‐ERBα transcription factors. REV‐ERBα reduces NFIL3 transcription, further repressing CD38. Meanwhile, the rhythmicity of expression is controlled by core clock components, and NAMPT regulates NAD^+^ synthesis. (B–D) The panels summarize three predicted feedback loops that are not fully verified. Loop 1 proposes that circadian regulation of NAMPT drives rhythmic NAD^+^ production, which tunes SIRT1 activity and, in turn, the circadian rhythm system (B). SIRT1, in turn, regulates the circadian rhythm system by deacetylating and modulating the NAMPT function of the CLOCK‐BMAL complex (B). Loop 2 proposes that PARP1 consumes NAD^+^ and that PARP1‐dependent modification of CLOCK impacts circadian timing (C). PARP1‐mediated PARylation of CLOCK modulates the circadian rhythm machinery, which, in turn, adjusts NAD^+^ levels via NAMPT, thereby establishing a second feedback loop (C). For feedback, the circadian clock regulates NAMPT transcription, thereby closing this loop (C). In the brain, loop 3 proposes that SIRT1 represses the CD38‐NFIL3‐REV‐ERBα axis, thereby stabilizing NAD^+^ levels and supporting robust circadian rhythm function (D). Loss of REV‐ERBα within the clock network suppresses CD38 via NFIL3, thereby altering NAD^+^ levels and forming a third feedback loop in the brain (D). BMAL1, brain and muscle Arnt‐like protein; CD38, cluster of differentiation 38; CLOCK, circadian locomotor output cycles kaput; NAD^+^, nicotinamide adenine dinucleotide; NAM, nicotinamide; NAMPT, nicotinamide phosphoribosyltransferase; NFIL3, nuclear factor, interleukin 3‐regulated; NR, nicotinamide riboside; NMN, nicotinamide mononucleotide; PARP1, poly(ADP‐ribose) polymerase 1; REV‐ERBα, reverse c‐erbAα; SIRT1, Sirtuin 1.

The circadian rhythm is systemically operated by negative feedback loops involving molecular subsets (Figure 1). In the core, circadian locomotor output cycles kaput (CLOCK) and brain and muscle Arnt‐like protein (BMAL1) form heterodimers and initiate the transcription of diverse downstream genes in a circadian fashion by binding to E‐box elements at the promoter region of target genes.^23^, ^24^, ^25^ CLOCK protein abundance and transcriptional activity are dynamically regulated after transcription by specific kinases, including casein kinase (CK) and post‐transcriptional modulators such as microRNAs.^26^, ^27^, ^28^ In addition, the CLOCK paralog neuronal Per‐ARNT‐Sim domain protein 2 (NPAS2) can functionally substitute for CLOCK, thereby preserving core oscillator integrity.^29^ The CLOCK–BMAL1 heterodimer activates circadian transcription of periods (PERs) and cryptochromes (CRYs) by binding E‐box elements within their promoters. Next, CRY1/2 directly docks on the CLOCK‐BMAL1 heterodimer – engaging the BMAL1 C‐terminal transactivation domain and the CLOCK PAS‐B core – to outcompete co‐activators and silence E‐box transcription, often while DNA binding is retained (Figure 1).^30^ PER alone does not measurably alter CLOCK‐BMAL1‐driven transcription.^31^ When PER is present with the CRY, the PER–CRY complex enters the nucleus and represses transcription by displacing CLOCK‐BMAL1 from promoters.^31^ Meanwhile, PER–CRY potentiates and phases repression by recruiting casein kinase 1δ/ε (CK1δ/ε) to the complex, which phosphorylates CLOCK and BMAL1.^30^ CK1δ/ε‐dependent phosphorylation converts promoter‐bound “quenching” into displacement of CLOCK‐BMAL1 from promoters, thereby defining the duration of the repression phase.^25^, ^30^, ^32^, ^33^ Together, these interactions create a core transcription‐translation feedback loop (TTFL) in which CLOCK‐BMAL1 activates PER and CRY, and the nuclear return of PER‐CRY complexes inhibits CLOCK‐BMAL1, thereby driving the 24‐h cycling oscillation (Figure 1).

Additionally, in the auxiliary loop, there is another TTFL between retinoid‐related orphan receptors (RORs) and reverse c‐erbAα, also referred to as nuclear receptor subfamily 1 group D member 1 (REV‐ERBα, also referred to as NR1D1), competing for the same ROR‐response elements (ROREs) in the *BMAL1* promoter, but they recruit opposite cofactors and enforce opposite chromatin states.^34^, ^35^, ^36^, ^37^ RORs bind ROREs as monomers via their zinc‐finger DNA‐binding domains and recruit coactivators (e.g., steroid receptor coactivator‐1 [SRC‐1]), promoting histone acetylation, open chromatin, and RNA Pol II loading – thereby increasing Bmal1 transcription.^38^, ^39^ REV‐ERBα/NR1D1 also binds ROREs and recruits co‐repressor complex, nuclear receptor corepressor‐histone deacetylase 3 (NCoR–HDAC3), which deacetylates nearby histones, compacts chromatin, and blocks Pol II initiation – reducing Bmal1 transcription.^35^, ^40^ Because ROR and REV‐ERBα/NR1D1 are themselves driven rhythmically (largely by BMAL1–CLOCK), their nuclear abundance and RORE occupancy oscillate across the day.^41^, ^42^ There is a paralogous nuclear receptor of REV‐ERBα/NR1D1, reverse c‐erbβ, also called nuclear receptor subfamily 1 group D member 2 (REV‐ERBβ, also referred to as NR1D2), a closely related paralog that recognizes many of the same RORE‐linked targets as REV‐ERBα/NR1D1 and can partially compensate when REV‐ERBα/NR1D1 activity is reduced, thereby helping maintain the rhythmic repressive arm of this auxiliary loop.^43^

Together, these TTFLs establish tissue‐specific circadian programs across the brain and periphery in a phase‐specific manner. The core circadian oscillator generates and stabilizes circadian oscillations and routes them to physiology.

### 1.3 Linking NAD^+^ pathways with circadian biology in dementia

NAD^+^ is an essential cellular cofactor that supports energy metabolism, redox homeostasis, stress responses, immune function, and circadian regulation. It also serves as a substrate for NAD^+^‐dependent enzymes, including sirtuins such as Sirtuin 1 (SIRT1), poly(adenosine 5'‐diphosphate‐ribose) polymerases such as poly(ADP‐ribose) polymerase 1 (PARP1), and cluster of differentiation 38 (CD38).^44^ A declining NAD^+^ level links to many of the hallmarks of aging and is likely an age‐related culprit of neurodegenerative diseases, including AD.^17^, ^45^, ^46^ Dementia models typically exhibit barriers to NAD^+^ biosynthesis and hyperexpression of NAD^+^‐consuming enzymes, leading to NAD^+^ depletion, and are accompanied by diverse biological symptoms.^47^ To overcome age‐associated dysfunctions, raising NAD^+^ levels can confer therapeutic benefits in dementia by acting through these pathways, and several NAD^+^‐modulating interventions are currently under clinical investigation.^17^, ^45^, ^48^ Detailed information on the metabolism, biology, its linkages to diseases due to depletion, and potential clinical applications is presented in recent reviews.^17^, ^45^

NAD^+^ metabolism and the circadian clock regulate each other. Cellular NAD^+^ levels oscillate and feed back onto clock machinery through enzymes that use or regenerate NAD^+^. Nicotinamide phosphoribosyltransferase (NAMPT), the rate‐limiting enzyme in the NAD^+^ salvage pathway, shows circadian expression that drives daily rhythms in NAD^+^ and thereby modulates the activity of SIRT1 and other NAD^+^‐dependent regulators of clock gene transcription.^49^, ^50^ Fluctuations in intracellular NAD^+^ remodel local chromatin structure and establish transcriptional feedback loops that sustain cell‐autonomous circadian oscillations. NAD^+^ enables deacetylation of core clock components such as BMAL1 and PER2 via SIRT1, a deacetylase, thereby modulating their stability, activity, and promoter binding.^13^, ^51^ Additionally, NAD^+^ is also required for PARP1‐dependent ADP ribosylation at clock gene loci, which reshapes chromatin architecture and influences clock‐controlled transcription.^52^, ^53^, ^54^ PARP1 activation by DNA damage consumes NAD^+^, and its activity can follow circadian patterns, so excessive or mistimed PARP1 signaling may blunt NAD^+^ oscillations and disturb clock‐controlled genomic maintenance.^52^, ^55^ CD38 is a major NAD^+^ hydrolase and membrane‐associated ectoenzyme that shows circadian expression in multiple tissues. Under inflammatory states, CD38 expression and activity increase, thereby dampening the amplitude of NAD^+^ oscillations and contributing to a progressive decline in cellular NAD^+^ levels.^56^, ^57^, ^58^, ^59^ Through these pathways, NAD^+^ and the circadian timing system form a bidirectional regulatory network (Figure 1).

Circadian rhythm disruption and the NAD^+^ pathway are reciprocally linked, and this coupling may remain amenable to intervention in dementia. Unlike previous reviews that focused primarily on circadian dysfunction and sleep disturbances in dementia, this review highlights how bidirectional interactions between NAD^+^ metabolism and circadian regulation operate across multiple dementia subtypes. We integrate evidence from clinical studies, animal models, and in vitro experiments and organize the discussion around the central pathological processes of dementia. We searched PubMed, Web of Science, Google Scholar, and ClinicalTrials.gov for relevant literature and trials published up to December 2025. This search was supplemented by manual screening of key articles and the reference lists of related reviews. Search terms included combinations of “NAD^+^” or “nicotinamide adenine dinucleotide” with terms such as “circadian rhythm,” “biological clock,” “sleep,” “cognition,” “dementia,” “Alzheimer's disease,” “Lewy body dementia,” “Parkinson's disease dementia,” “frontotemporal dementia,” “vascular cognitive impairment,” “SIRT1,” “PARP1,” “CD38,” and “NAMPT.” Eligible studies included original English language articles, clinical studies, animal studies, mechanistic cellular studies, and reviews that directly examined the relationships among NAD^+^ biology, circadian rhythm regulation, sleep disturbances, and dementia or related neurodegenerative conditions associated with cognitive decline. We excluded studies that did not address these core themes, provided insufficient evidence, or offered limited translational relevance. Priority was given to recent studies and seminal work that established the conceptual basis for this review. On this basis, we distinguish mechanisms supported by direct evidence from those that remain inferential, highlight testable hypotheses with translational relevance, and identify subtype‐specific molecular nodes that may guide future intervention studies.

## 2 CIRCADIAN RHYTHM DISORDERS IN DEMENTIA

Although light is obviously the strongest stimulus to operate our physiological systems, the human body has an endogenous circadian system and physiological strategies to keep the clock. In dementia, some patients show a progressive phase advance of circadian rhythms, reduced amplitude of core clock gene expression, and diminished responsiveness to external zeitgeber time (ZT) such as light and feeding schedules.^60^, ^61^ These alterations compromise the robustness of circadian oscillations in central and peripheral clocks, leading to an advanced timing of sleep‐wake cycles, hormonal secretions, and metabolic processes.^7^, ^61^, ^62^ The attenuated amplitude arises from circadian‐related molecular changes, including decreased expression and post‐translational modifications of clock proteins, impaired NAD^+^‐dependent sirtuin activity, and reduced sensitivity of photic and metabolic inputs to the SCN.^61^ Consequently, the weakened entrainment to environmental cues leads to the fragmentation of behavioral rhythms and reduced synchronization across tissues, exacerbating metabolic dysregulation, cognitive decline, and vulnerability to neurodegenerative pathology (Table 1). Interventions that restore ZT strength or enhance molecular clock function hold promise for mitigating these circadian deficits inherent in dementia.

**TABLE 1:** Altered core clock genes across mechanisms of dementia.

Reasons of cognitive decline	Model(s)	Changes	Functional outcomes	Reference
Aging	Young versus old hamster brain, multiple extra SCN oscillators including memory‐related regions	BMAL1 mRNA level↓ with age in SCN regions, and time of day pattern also altered	Suggests weakened local clocks outside the SCN that can impact memory‐relevant circuits	Chen et al., 2016^63^
AD	Human pineal gland from controls, preclinical AD, clinical AD	BMAL1, PER1, CRY1 rhythmic mRNA oscillation lost in AD groups	Loss of pineal molecular rhythms can contribute to disrupted melatonin timing and sleep and wake disturbances	Duncan et al., 2013^64^
AD	Human cohorts, early stage of AD, epigenetic time series framework	BMAL1 shows aberrant cycles of DNA methylation linked to circadian alterations in early AD	Supports clock dysregulation early in disease course	Wu et al., 2006^65^
AD	5xFAD mouse model and cellular systems with amyloid beta exposure	BMAL1 and PER2 protein amplitude↓ in AD group	Circadian rhythm disruption and neurodegeneration‐related phenotypes	Cronin et al., 2017^66^
AD spectrum tauopathy	Tg4510 tauopathy mouse model with hypothalamus, SCN, hippocampus analyses	PER2 cyclic expression↓ in hypothalamus, PER2 and BMAL1 cyclic expression↓ in hippocampus	Lengthened free‐running period and disrupted circadian behavior when tauopathy is present	Stevanovic et al., 2017^67^
PD	Human PBMC, plasma, large case control cohort	mRNA level of BMAL1↓, CLOCK ↓, CRY1 ↓, PER1 ↓, PER2 ↓ in PD	Lower clock gene expression associates with sleep and wake disturbances, including probable RBD and daytime sleepiness	Li et al., 2021^68^
PD	MPTP‐induced mouse model, substantia nigra time series across the day	REV‐ERBα mean level↓ and diurnal oscillation lost, BMAL1 and PER2 amplitude slightly ↓	Links disrupted clock regulation with microglial activation and neuroinflammation in PD model	Kou et al., 2022^69^
FTD spectrum TDP‐43 proteinopathy	Mouse TDP‐43 knockdown in brain	BMAL1, CLOCK, CRY1, PER2 expression altered, BMAL1 ubiquitination ↑	Impaired autonomous circadian behavior plus cognitive and balance deficits	Gu et al., 2025^70^
Stroke related cognitive impairment risk (may induce VCID)	Acute ischemic stroke patients, diurnal blood expression profiling	REV‐ERBα and PER1 show differences in specific time compared with controls	Suggests partial preservation but altered timing features of peripheral clock outputs after stroke	Pajediene et al., 2022^71^
Vascular hypoxia stress (may induce VCID)	Mouse intermittent hypoxia model	BMAL1 and Rev‐erbα mRNA level amplitude↓ in brain, mRNA level of PER1, Clock, Rev‐erbα↓ in liver	Supports a route by which vascular oxygen instability can disrupt molecular clocks relevant to cognition	Koritala et al., 2021^72^

Abbreviations: 5xFAD, five familial Alzheimer's disease mutations; AD, Alzheimer disease; BMAL1, brain and muscle Arnt‐like protein; CLOCK, circadian locomotor output cycles kaput; FTD, frontotemporal dementia; MPTP, 1 methyl 4 phenyl 1,2,3,6 tetrahydropyridine; PBMC, peripheral blood mononuclear cell; RBD, REM sleep behavior disorder; SCN, suprachiasmatic nucleus; TDP‐43, TAR DNA binding protein 43; VCID, vascular cognitive impairment and dementia.

### 2.1 Synucleinopathies

Synucleinopathies, including Lewy body dementia (LBD), Parkinson's disease (PD), and multiple system atrophy (MSA) are characterized by neuronal α‐synuclein inclusions, and ∼70% of nigrostriatal neurons have already died by the time the disease is diagnosed.^73^ Clinical observation indicates that more than 70% of individuals with LBD exhibit rapid eye movement sleep behavior disorder (RBD) at early diagnosis, and RBD can precede the onset of symptoms in PD and LBD by more than a decade.^74^ RBD is increasingly recognized as a core diagnostic and prognostic feature of LBD and is considered a potential biomarker of preclinical disease to enable early intervention strategies that delay or attenuate disease progression.^75^, ^76^

Sleep comprises four stages – rapid eye movement (REM) sleep and three non‐REM (NREM) stages – collectively referred to as sleep macro‐architecture. Elderly people have shown disrupted sleep habits, with shorter duration and increased fragmentation, due to diverse factors including hormonal fluctuations, changes in circadian rhythm, collapse of the upper airways caused by obstructive sleep apnea (OSA), and lifestyle factors.^77^, ^78^, ^79^ The master clock region, SCN, provides high‐amplitude temporal cues that stabilize REM timing via projections to the subcoeruleus (SLD) and the pedunculopontine/laterodorsal tegmental nuclei.^80^ In RBD, dampened SCN output, blunted nocturnal melatonin rhythms, and reduced dopaminergic oscillatory amplitude weaken this hierarchical structure. The consequence is that patients show unstable REM to NREM transitions and loss of motor suppression during REM sleep. Brainstem nuclei involved in REM muscle inhibition commonly exhibit rhythmic expression of BMAL1, CLOCK, PER, and CRY, which modulate synaptic excitability and inhibitory tone across the 24‐h cycle.^81^ However, neuroinflammatory stress and early α‐synuclein accumulation attenuate these transcriptional‐translational feedback loops, degrading circadian precision at the cellular level.^82^

Anatomically, the pontine or medulla is heavily involved in the circuitry of the REM process muscles, and GABAergic ventromedial medulla (VMM) degeneration seems to specifically affect the RBD region more than the SLD region, which has been proposed as the hot spot for RBD.^83^ Kashiwagi et al. reported that corticotropin‐releasing hormone‐binding protein (CRHBP^+^)‐expressed neurons in the pontine sublateral‐dorsal tegmentum (SubLDT) regulate REM state depending on the projection site, gastrointestinal neuron or basal forebrain neuron, and their ablation‐induced RBD‐like phenotype. PD patients show a low population of CRHBP+ neurons and correlate with pSyn inclusion, suggesting insight into the therapy underlying sleep dysfunction‐related neurodegenerative disease.^84^ On the contrary, the molecular clock system is barely investigated in this area, although those regions are considered a critical part that sends descending projections to the spinal cord to modulate the sleep state, particularly REM. Per1 was the only clock gene that was measured in locus coeruleus (LC) from mice and Syrian hamster models, and Per1::Luc mice did not show the promoter‐driven luciferase activity in LC when Syrian hamsters presented it across the day.^85^

A concern is the role of the NAD^+^‐dependent sirtuins and PD. For example, a causal relationship between compromised NAD^+^/sirtuin activity and circadian disturbance in PD pathology remains unestablished. It is unclear whether sirtuin dysfunction is an upstream driver of instability in the core molecular clock (including altered circadian rhythm‐related gene rhythmicity and dysregulated REM and non‐REM sleep state transitions) or whether primary circadian disruption secondarily impairs sirtuin function. Addressing these gaps will require studies that integrate circadian with NAD^+^ in PD.

### 2.2 Alzheimer's disease (AD)

AD, the most common form of dementia, is characterized by amyloid plaques and tau tangles, which drive neurodegeneration in memory‐related brain regions such as the hippocampus and parietal cortex, leading to cognitive decline.^86^, ^87^ Sleep and circadian rhythm disturbances are prevalent across the AD spectrum and often emerge early, as evidenced by polysomnography and actigraphy showing reduced sleep efficiency, increased nocturnal awakenings, and greater sleep‐wake fragmentation.^9^, ^88^, ^89^, ^90^, ^91^ During the preclinical stage, cognition is largely preserved despite detectable amyloid pathology, while circadian disruption is already present and correlates with amyloid burden.^18^ Prospective studies further suggest that poorer sleep quality and reduced circadian robustness precede and predict subsequent amyloid accumulation, consistent with their potential value as early biomarkers and as contributing risk modifiers.^92^ In the mild cognitive impairment (MCI) stage, disturbances become more prominent, which includes reduced sleep efficiency, more wake after sleep onset, and decreases in slow wave sleep and rapid eye movement sleep.^93^, ^94^ In the dementia stage, sleep and circadian disruption are typically most severe, characterized by marked fragmentation, increased daytime napping, reduced rhythm amplitude with phase shifts, and nocturnal agitation.^12^, ^95^ Systematic reviews and meta‐analyses consistently report broad deterioration in total sleep time, sleep efficiency, and slow‐wave and REM sleep.^12^

Mechanistically, neurodegeneration can disrupt suprachiasmatic nucleus signaling, reduce melatonin‐related synchrony, weaken entrainment to light and other ZTs, and impair sleep consolidation.^96^, ^97^ Evidence also supports bidirectionality: experimental sleep deprivation or selective disruption of slow‐wave sleep increases soluble amyloid beta (Aβ).^98^, ^99^ At the same time, human studies associate a higher amyloid burden with reduced slow‐wave activity and poorer memory consolidation.^100^ Chronic disruption may further compromise perivascular clearance, thereby promoting the accumulation of amyloid and tau.^101^, ^102^ Consistent with the potential for intervention, Weidong Le group reported that long‐term exercise restored abnormal diurnal hyperactivity and improved sleep‐wake architecture in AD rodents, as assessed by electroencephalogram (EEG), including reduced wakefulness and less fragmented REM sleep, and attenuated pathology, including memory deficits, amyloid plaques, phosphorylated tau accumulation, and neuroinflammation.^89^ At the molecular level, AD is linked to altered core clock circuitry involving BMAL1, CLOCK, PER, and CRY, with additional regulation by REV‐ERB and ROR, and experimental models indicate Aβ‐associated reductions in BMAL1 activity and damped PER2 rhythms.^103^, ^104^ Clock system disruption may also exacerbate neuroimmune dysfunction by reshaping diurnal complementary programs under BMAL1 and REV‐ERB control and by altering microglial synaptic phagocytosis.^21^ In addition, reports link REV‐ERBα/NR1D1 to brain NAD^+^ regulation via interleukin 3‐regulated (NFIL3)/CD38 pathway, which can influence tau‐related pathology in mice.^105^

Most existing studies emphasize how circadian clocks shape NAD^+^ metabolism, whereas the reciprocal regulation of circadian timing by NAD^+^ in AD has received far less attention. In aging animal models, NAD^+^ augmentation can strengthen circadian rhythmicity and restore rhythmic gene expression through SIRT1‐dependent mechanisms. In AD mouse models, NAD^+^ supplementation has also been reported to improve cognition via multiple pathways, including enhanced mitophagy and attenuation of neuroinflammation. In addition, it suggests that targeting circadian regulation may represent a potentially actionable mechanism, further supporting the application of NAD^+^ in AD.

### 2.3 Frontotemporal dementia (FTD)

Patients with FTD often show long sleep time but present advanced or fragmented activity patterns, reflecting impaired central circadian pace making and weakened synchronization between behavioral outputs and peripheral metabolic cues.^106^ Zhang et al. showed the pathological hallmark protein of FTD/amyotrophic lateral sclerosis (ALS), transactive response DNA‐binding protein 43 (TDP‐43), rhythmically expressed in SCN and its ablation led to dysfunction of intracellular arrhythmicity, transcriptional activation regulation, and clock gene expression, as well as severe behavioral defects such as locomotor activity and depression‐like behavior.^107^ These circadian abnormalities contribute to metabolic dysregulation, including altered glucose metabolism and energy homeostasis, which are linked to the behavioral phenotypes of FTD.

Behavioral variant FTD (bvFTD) is characterized by pronounced disorganization of behavioral rhythms, including disrupted sleep‐wake cycles and severely altered mealtime behaviors, which collectively exacerbate metabolic and neuropsychiatric symptoms.^108^, ^109^ Abnormal eating behaviors are present in over 80% of bvFTD patients and represent a core diagnostic feature, with distinctive manifestations including hyperphagia. As meal timing serves as the most powerful synchronizing signal for peripheral metabolic clocks, more potent than the light‐dark cycle, dysregulated eating disrupts the meal‐clock‐transcriptome synchronization, flattening hepatic clock gene amplitude and impairing glucose and lipid metabolism in FTD. NAD^+^‐dependent hypothalamic SIRT1 in appetite‐regulating neurons integrates feeding signals with circadian timing through NAD^+^‐driven oscillations in the arcuate nucleus.^110^, ^111^ Disrupted meal timing in FTD attenuates hypothalamic NAMPT expression, impairing SIRT1's regulation of appetite‐suppressing pathways and clock protein deacetylation, thereby driving uncontrolled hyperphagia. Misaligned feeding also breaks the feed‐entrained oscillator circuit, preventing food‐driven synchronization of REV‐ERBα/NR1D1 and other hypothalamic circadian regulators that maintain NAD^+^ pools. Thus, there is a potential pathogenic spiral shown in FTD that awaits further validation: dysregulated feeding → suppressed hypothalamic NAD^+^ and sirtuin activity → impaired peripheral metabolic clock synchronization → neuroinflammation and neurodegeneration, establishing that meal‐circadian‐molecular dysfunction may be a critical therapeutic nexus in FTD pathophysiology. In this context, addressing meal timing and reinforcing circadian entrainment represent promising therapeutic avenues to improve quality of life and slow disease progression in FTD patients.

### 2.4 Vascular contributions to cognitive impairment and dementia (VCID)

VCID is the second most common dementia type and demonstrates more progressive cognitive decline compared to other dementia forms. Large‐scale studies have shown that VCID is frequently complicated by OSA and nocturnal hypoxia, conditions that profoundly impair circadian regulation and exacerbate cognitive decline.^112^, ^113^, ^114^ Repetitive airway collapse also disrupts both the central circadian rhythm in the SCN and peripheral clock synchronization across tissues.^115^ Repetitive airway collapse also disrupts both central circadian peacemaking in the SCN and peripheral clock synchronization across tissues. This OSA‐induced perturbation impairs SCN function through multiple mechanisms: (1) reduced expression of core clock genes (BMAL1, CLOCK, PER1, PER2) driven by hypoxia‐responsive microRNAs (particularly miRNA‐181a) and (2) suppression of NAD^+^ biosynthesis, thereby weakening NAD^+^‐dependent SIRT1 deacetylase activity and promoting downstream neuroinflammation and oxidative stress – key drivers in both VCID and LBD pathophysiology. Specifically, OSA‐induced circadian clock disruption dysregulates the expression of BMAL1‐dependent SIRT1 transcription, leading to persistent elevation of NADPH Oxidase 4 (NOX4)‐mediated reactive oxygen species (ROS) production in vulnerable neural and vascular tissues. Additionally, OSA increases inflammatory cytokine secretion (TNFα, IL‐6, IL‐8) and impairs their normal circadian rhythmicity, which compounds neuroinflammatory damage in susceptible brain regions.

Clinical studies demonstrate that OSA prevalence and severity correlate with worse cognitive outcomes, accelerated neurodegeneration, and more pronounced circadian rhythm disturbances in VCID and mixed‐dementia populations.^116^, ^117^ Longitudinal cohort studies document that nocturnal hypoxemia (prolonged time at SpO_2_ < 90%) predicts steeper decline in global cognition, memory, processing speed, and executive function – domains particularly vulnerable in VCID. Therapeutic interventions targeting OSA, including continuous positive airway pressure (CPAP), have demonstrated significant improvements not only in sleep quality and architecture but also in the stabilization of circadian rhythms and slowing of cognitive decline in patients with MCI and OSA.^118^ In patients, long‐term CPAP therapy (≥13 months sustained use) provides protective effects on cognitive performance by stabilizing the SIRT1 and reducing OSA‐driven oxidative stress and circadian misalignment, with sustained improvements in episodic memory, processing speed, and executive function.^119^, ^120^ OSA and the associated nocturnal hypoxia are key modifiable risk factors for VCID and may accelerate dementia progression by promoting the production of intermittent hypoxia‐driven ROS generation, dysregulation of circadian clock genes, impairment of the NAD^+^‐SIRT1 signaling axis, and systemic neuroinflammation. However, it remains unclear whether targeting the NAD^+^‐SIRT1 pathway can ameliorate inflammation and circadian rhythm disturbances in this setting. Additionally, OSA also promotes inflammatory activation, and CD33, an immune regulator linked to NAD^+^ consumption, may further reduce NAD^+^ availability, with evidence that CD33 interacts with hypertension to influence brain and cognitive aging. Thus, it is important to determine whether OSA‐driven CD33 changes contribute to NAD^+^ depletion and circadian disruption, supporting NAD^+^ as a potential circadian‐based therapeutic target in VCID.

## 3 CIRCADIAN ALTERATIONS AND POTENTIAL MECHANISMS OF SIRT1, PARP1, AND NAMPT IN DEMENTIA

Multiple lines of evidence suggest a linkage among the circadian clock, NAD^+^ metabolism, and neurodegenerative disease. NAD^+^ acts as a clock‐gated metabolite because it fuels sirtuins and PARPs, which modify core clock proteins and chromatin to shape rhythm amplitude, phase, and network synchrony. At the same time, based on preclinical research, NAMPT‐dependent NAD^+^ salvage is under circadian control, creating a feedback loop in which disease‐related NAD^+^ depletion or increased NAD^+^ consumption dampens circadian output, exacerbating sleep disruption and cellular stress. Conversely, circadian disruption can reduce NAMPT expression and function, further impairing NAD^+^ synthesis. Here we will review several of these mechanisms to explore this complex interaction and identify therapeutic opportunities.

### 3.1 SIRT1 rhythms in dementia

SIRT1 is a class III lysine deacetylase whose catalytic activity requires NAD^+^ as a co‐substrate. During the deacetylation reaction, SIRT1 removes an acetyl group from an acetylated lysine residue (Ac‐Lys) and transfers it to NAD^+^, generating deacetylated lysine (Lys), nicotinamide, and a new product, 2′‐O‐acetyl‐ADP‐ribose (overall: Ac‐Lys + NAD^+^ → Lys + nicotinamide + 2′‐O‐acetyl‐ADP‐ribose).^121^ SIRT1 activity tracks NAD^+^ availability and the NAD^+^/NADH (nicotinamide adenine dinucleotide) ratio. Moreover, sustained elevations of nicotinamide inhibit SIRT1 via base exchange, so low NAD^+^ or high nicotinamide inhibits deacetylation.^122^, ^123^ Together, NAD^+^ and its metabolites form a feedback loop with SIRT1, establishing functional interdependence between them.

SIRT1 levels decline in dementias, where circadian and sleep disturbances are prevalent. As SIRT1 modulates core clock components, it presents a promising therapeutic target for restoring circadian regulation in dementia.^124^, ^125^, ^126^

#### 3.1.1 Effects of SIRT1 on BMAL1 and PER2 deacetylation

Core clock coupling in mammalian circadian systems is orchestrated by an integrated network, centrally involving NAD^+^‐dependent sirtuin (SIRT1/SIRT3) regulation of BMAL1 and PER2 deacetylation.^51^, ^127^ The periodic activation of SIRT1 thus synchronizes chromatin remodeling with circadian gene expression, as SIRT1 also targets core histones (e.g., H3K9, H4K16), shaping epigenetic landscapes to ensure temporally precise genome accessibility and transcriptional amplitude. CLOCK functions as an intrinsic acetyltransferase that acetylates BMAL1,^128^ whereas SIRT1, acting as a deacetylase, binds the CLOCK‐BMAL1 transcriptional complex and promotes BMAL1 deacetylation.^13^, ^128^ Generally, acetylated BMAL1 (acBMAL1) promotes CRY recruitment to the E‐box promoter element, which downregulates CLOCK‐BMAL1‐driven transcription and helps set the circadian period and amplitude.^129^ This deacetylation of SIRT1 regulates acetyltransferase activity of CLOCK on BMAL1, limiting excessive transcription of Per and Cry genes and thereby modulating downstream oscillators.^13^, ^128^ SIRT1 also binds PER2 in a circadian oscillation, facilitates its deacetylation and proteasomal degradation, and further accelerates translocation of PER2.^51^ Faster PER2 turnover shortens the interval of repression on CLOCK‐BMAL1 activity and helps initiate the next activation phase.^130^, ^131^

During aging, declining SIRT1 expression in the SCN weakens CLOCK and BMAL1 signaling and influences the acetylation of BMAL1 and PER2, thereby lengthening the intrinsic circadian period, dampening rhythm amplitude, and impairing re‐entrainment capacity.^132^ Brain‐specific SIRT1 overexpression partially alleviates these deficits in mice.^13^, ^14^, ^15^ In a 6‐OHDA‐induced PD rat model, the SIRT1 activator, resveratrol, decreased BMAL1 acetylation and limited its association with CRY1, thereby reversing 6‐OHDA‐related impairment of antioxidant activity.^133^ Although direct demonstrations to support SIRT1‐mediated control of circadian rhythms in dementia are limited, convergent evidence indicates an indirect effect. Time‐restricted feeding (TRF) activates SIRT1 and improves pathological phenotypes and cognitive impairment in AD and PD mouse, as well as circadian rhythm disruption in AD mouse, suggesting that TRF may alleviate circadian disturbances in AD through SIRT1 signaling.^134^, ^135^, ^136^ Meanwhile, resveratrol modulates circadian rhythms across multiple disease systems.^137^, ^138^ It also confers neuroprotection in AD, PD, and other tauopathy mouse models, improving disease phenotypes.^139^ However, NAD^+^, which declines in both dementia and aging, availability governs SIRT1 activity, which in turn controls protein acetylation. In mice, depletion of NAD^+^ despite intact SIRT1 causes hyperacetylation of PER, indicating that reduced NAD^+^ directly limits SIRT1‐mediated deacetylation of circadian proteins.^14^ This implies that motivating translational efforts with NAD^+^‐boosting and sirtuin‐activating compounds for clock and health restoration is important. Concurrently, dementia is associated with circadian rhythm disturbances, including altered BMAL1 rhythmicity across multiple brain regions, yet it remains unclear whether enhancing the NAD^+^‐SIRT1 axis can restore these rhythms by promoting SIRT1‐mediated deacetylation of BMAL1. Although direct evidence is still lacking, existing studies suggest that NAD^+^‐SIRT1‐BMAL1 is a plausible mechanistic node connecting dementia with rhythm disturbances.

#### 3.1.2 SIRT1/SIRT3 and AMP‐activated protein kinase (AMPK)

Sleep homeostasis reflects how sleep pressure builds during wakefulness and wanes during sleep, and it is tightly coupled to synaptic plasticity and cellular energy state through NAD^+^‐dependent SIRT1 and the energy sensor AMPK. Oscillations in NAD^+^ availability tune SIRT1 activity, thereby shaping slow‐wave activity and the gene programs that adjust synaptic strength and circadian timing. SIRT1 is also strongly expressed in wake‐related neuronal populations, including those in the LC, linking metabolic state to REM and wake regulation, and its deficiency severely reduces total wake time in the hyper‐REM state without altering NREM across the day.^140^ A recent study showed that the small‐molecule enhancing SIRT1 activity, DDL‐218, verified its role in apolipoprotein E (APOE ε4)‐expressing neuronal cells and enhanced memory‐related behavior in APOE ε4‐positive 5xFAD AD mice upon administration of DDL‐218.^141^ These support that SIRT1 activation may be an effective strategy to mitigate sleep disturbances and memory impairment in AD. However, whether circadian regulation causally mediates the cognitive benefits of SIRT1 activation remains to be determined. Concurrently, AMPK acts as a cellular energy sensor that synergizes with SIRT1 to adapt neuronal bioenergetics to fluctuating sleep‐wake states, promoting mitochondrial biogenesis and metabolic homeostasis essential for synaptic efficacy.^142^, ^143^ Long‐term sleep deprivation or intermittent hypoxia, as in OSA, one of the important causes of circadian rhythm disruption in VCID, impairs AMPK pathway by reducing antioxidants, including SIRT1, nuclear factor erythroid 2‐related factor 2 (NRF2), and glutathione S‐transferase alpha 3 (GSTA3).^144^, ^145^

SIRT3 is another NAD^+^‐dependent class III lysine deacetylase. It plays a pivotal role in sustaining neuronal resilience during sleep by preserving mitochondrial integrity, limiting oxidative stress, and maintaining efficient ATP production by regulating numerous cell types. Cortical mitochondrial SIRT3 knockout mice showed hyper‐subcellular Ca^2+^ levels, leading to the inhibition of the AMPK/peroxisome proliferator‐activated receptor‐gamma coactivator 1‐alpha (PGC‐1α),^146^ and recent findings propose that dopamine‐D2‐agonists could regulate this axis by blocking Cytochrome C in mitochondria.^147^ SIRT3 ensures that the substantial bioenergetic demands of synaptic plasticity are met, thereby safeguarding cognitive function against the adverse effects of sleep deprivation and aging. Taken together, NAD^+^‐SIRT1/AMPK signaling and mitochondrial SIRT3 may form a coupled network that integrates sleep homeostasis with energy metabolism and synaptic remodeling, offering mechanistic insight.

Substantial evidence links NAD^+^ to cellular energy homeostasis and to circadian regulation, in part through SIRT1‐mediated control of core clock components. However, the mechanistic relationships among NAD^+^, energy homeostasis, and the circadian system remains incompletely defined. It is unclear whether this coupling is bidirectional and mutually dependent, including whether the benefits of NAD^+^ augmentation depend on the circadian rhythm system and whether the dosing time of NAD^+^ can reshape daily rhythms in bioenergetic output. In AD, mitochondrial dysfunction and circadian disruption often co‐occur, suggesting that restoring NAD^+^ could improve both processes through shared pathways. These hypotheses remain to be tested and will require studies that integrate circadian timing, metabolic readouts, and dementia‐relevant phenotypes.

### 3.2 Rhythms of PARP1 activity in dementia

PARP1 participates in DNA damage repair, maintenance of genomic stability, cell proliferation, differentiation, and apoptosis.^148^ During activation, PARP1 can consume large amounts of NAD^+^, and persistent DNA damage or chronic PARP1 signaling can deplete cellular NAD^+^ and ATP, causing energy failure and cell death. In dementia and neurodegenerative diseases, accumulated DNA damage, oxidative stress, and chronic neuroinflammation lead to overactivation of PARP1 and subsequent NAD^+^ depletion.^148^, ^149^, ^150^, ^151^ PARP1 activity is elevated in AD and PD patients and is stimulated by the accumulation of Aβ plaques and α‐syn aggregates in the brain.^148^, ^151^, ^152^, ^153^ In mouse models and cells, activated PARP1 promotes Aβ deposition and α‐syn aggregation, thereby worsening clinical and pathological features.^151^, ^154^ Consistent with this, Parp1‐deficient mice show reduced Aβ‐induced microglial activation and α‐syn degradation via autophagy.^151^, ^154^, ^155^ Neurovascular coupling is impaired in aged mice, and treatment with the PARP inhibitor PJ 34 improves aortic ring vasodilation, restores cerebral microvascular function, and enhances spatial memory.^156^ Multiple studies have also shown that PARP inhibitors attenuate ischemic brain injury after stroke, which can induce vascular dementia, and some have further reported neuroprotective effects in animal models.^157^ In TDP‐43 proteinopathies, PARP1 facilitates TDP‐43 liquid‐liquid phase separation and promotes stress granule assembly, whereas inhibition of PARP reduces TDP‐43‐associated neurodegeneration in *Drosophila*.^158^ Moreover, in *Chromosome 9 Open Reading Frame 72 (C9orf72) Drosophila* model of FTD and ALS, loss of PARP1 activity inhibits stress granule formation and suppresses neurodegeneration.^159^ Interestingly, PARP1 inhibition increases cellular NAD^+^ levels and enhances SIRT1 activity. Meanwhile, as a deacetylase, SIRT1 thereby modulates PARP1 function by deacetylating.^53^, ^160^, ^161^

PARP1 also modulates the circadian rhythm system. PARP1 binds the CLOCK‐BMAL1 complex and directly PARylates CLOCK, which disrupts CLOCK binding to BMAL1 target DNA and alters its interactions with PER and CRY.^162^ Meanwhile, PARP1 activity also shows oscillatory expression for 24 h in the mouse liver, influenced by food intake.^162^, ^163^ Since PARP1 requires NAD^+^, this activity has been proposed to reflect cellular NAD^+^ availability.^162^, ^163^ Although NAD^+^ levels exhibit circadian oscillation in mammals, it does not fully account for the circadian activity of PARP1 in vivo, as an excess of NAD^+^ does not alter PARP1 rhythmicity in vitro.^162^ In the context of sleep, sleep loss induces DNA damage, and following sleep deprivation, PARP1 activity, a DNA damage sensor, increases in both humans and animal models.^164^ Inhibition of PARP1 activity reduces sleep‐dependent chromosome dynamics and repair, whereas PARP1 promotes neuronal DNA repair and supports the homeostatic drive for sleep in animal models.^53^, ^164^, ^165^

Although no studies have directly demonstrated how PARP1 alters circadian rhythms in dementia, multiple lines of evidence point to a mechanistic link.^148^, ^166^ In dementias, increased DNA damage elevates PARP1 activity, accelerating NAD^+^ consumption, contributing to NAD^+^ decline, and may impair NAD^+^‐dependent circadian regulation. Previous studies suggest that elevated PARP1 can promote sleep by supporting DNA repair, but in the Aβ *Drosophila* model, increased PARP1 is instead associated with reduced sleep.^53^, ^164^, ^165^, ^167^ Sleep and circadian disruption are complex phenotypes influenced by multiple pathways. Acute PARP1 activation may enhance sleep through its role in repairing DNA damage, but prolonged PARP1 activation may have more complicated consequences, which requires further investigation.^164^ Meanwhile, SIRT1 suppresses PARP1 activity via deacetylation and can reduce PARP1 expression through promoter repression, particularly under stress.^53^, ^168^ Thus, NAD^+^ supplementation may modulate PARP1 via SIRT1, but direct evidence of this remains limited. Therefore, the relationship between PARP1 and circadian rhythms in dementia warrants deeper investigation. Changes in PARP1 abundance alone may not be sufficient to become the treatment target of dementia. Preserving molecular homeostasis and limiting downstream harm may be more informative. A controlled reduction in PARP1 activity, together with NAD^+^ repletion, could, in principle, maintain PARP1‐mediated DNA repair while supporting SIRT1‐dependent circadian regulation. However, this hypothesis requires direct experimental validation and further mechanistic studies.

### 3.3 NAMPT rhythms in dementia

NAMPT is a key rate‐limiting enzyme that maintains NAD^+^ levels. In mammals, NAD^+^ biosynthesis occurs primarily through four pathways, of which the salvage pathway is the major source.^17^, ^45^ In this pathway, NAMPT catalyzes the rate‐limiting conversion of nicotinamide to nicotinamide mononucleotide (NMN), which is then rapidly converted to NAD^+^. Accordingly, NAMPT activity is a key determinant of NAD^+^ resynthesis rate, and adequate NAMPT function is required to maintain NAD^+^ levels sufficient for processes such as energy metabolism.^57^, ^169^

However, NAD^+^ pathway homeostasis is not solely dependent on NAMPT in all contexts. In fat‐specific NAMPT‐deficient mice (FANKO), continuous NAD^+^ precursor infusion restored NAD^+^ in brown adipose tissue (BAT) and epididymal white adipose tissue (eWAT), rescuing circadian gene expression and metabolic phenotypes.^170^ Consistently, recent evidence suggests that astrocytic REV‐ERBα/NR1D1 regulates NAD^+^ tone by modulating CD38 expression and NAD^+^ consumption in tauopathy mouse models rather than the NAMPT biosynthesis pathway described in heart and liver.^105^, ^171^

In dementia animal models, hippocampal NAMPT expression and NAD^+^ levels decline, and experimental knockdown of NAMPT in hippocampal circuits induces cognitive deficits, supporting a causal link between reduced NAMPT‐dependent salvage and neuronal vulnerability.^17^ NAMPT is central to NAD^+^ metabolism in dementia, but the strength of evidence differs by diagnosis. In AD models, NAMPT and NAD^+^ levels are reduced in the cortex and hippocampus of APP/PS1 mice, and restoring this pathway ameliorates pathology.^172^ PD mouse models show that targeted loss of NAMPT in substantia nigra dopaminergic neurons is sufficient to cause neuronal degeneration and Parkinson‐like motor deficits, demonstrating a cell‐autonomous requirement for NAMPT to sustain NAD^+^ and neuronal survival in the nigrostriatal system.^173^ Circulating NAMPT has also been proposed as a potential blood biomarker in PD by integrative transcriptomic meta‐analysis in PD patients.^174^ In vascular dementia, the evidence remains largely circumstantial, but diverse cerebrovascular injury models reveal a protective role for NAMPT activity during ischemia and reperfusion, maintaining energy homeostasis while mitigating inflammatory injury.^175^

The *NAMPT* gene showed powerful diurnal rhythmicity following NAD^+^ output in tissues such as the liver (primary NAD^+^ rhythm source), skeletal muscle, heart, and adipose tissues in animal models with cell type‐specific resolution of the underlying molecular circuitry.^14^, ^49^, ^170^, ^176^ The promoter of *NAMPT* contains E‐box elements that are rhythmically bound by the CLOCK‐BMAL1 heterodimer in a promoter‐dependent manner,^176^ driving circadian transcription of NAMPT and thereby diurnal fluctuations in intracellular NAD^+^ levels.^49^ These findings suggest that NAMPT regulation is a key determinant of alterations in NAD^+^ levels and circadian rhythms in dementia and is directly controlled by the circadian rhythm system. Meanwhile, SIRT1 associates with the CLOCK‐BMAL1 complex and is recruited to the E‐box in a time‐dependent manner that tracks CLOCK‐BMAL1 occupancy.^13^ Thus, CLOCK and SIRT1 jointly regulate circadian chromatin remodeling at the NAMPT promoter. Since NAMPT controls NAD^+^ synthesis, intracellular NAD^+^ levels directly modulate the deacetylase activity of SIRT1, and SIRT1 can influence acBMAL1 and downstream function, these interactions form an enzyme transcription feedback loop (Figure 1). Although direct evidence remains limited, reduced NAMPT activity can be predicted to disrupt NAD^+^ homeostasis and further impair downstream circadian regulation. Altered expression or function of core clock components can also disrupt or promote the circadian oscillation of NAMPT. In dementia, modulation of circadian rhythms or NAMPT expression may strengthen this regulatory loop, thereby alleviating circadian disruption and associated cognitive impairment.

### 3.4 Mitochondrial ATP production rhythm in dementia

Continuous ATP production is required to support synaptic transmission, axonal transport, protein quality control, and global protein homeostasis, all of which progressively decline in dementias such as AD. Mitochondrial ATP output decreases in dementia, leading to energetic insufficiency, elevated oxidative stress, and increased neuronal vulnerability, further inducing cognitive decline.^17^, ^177^, ^178^ ATP levels in the brain exhibit circadian oscillations, indicating that cellular energy availability is under clock control.^179^, ^180^ NAD^+^ is reduced to NADH during the tricarboxylic acid (TCA) cycle, and NADH is subsequently oxidized back to NAD^+^ by the electron transport chain, thereby supporting oxidative phosphorylation and ATP production. Because NAD^+^ availability can be rate‐limiting, both NAD^+^ abundance and the NAD^+^ to NADH ratio are important determinants of mitochondrial energy efficiency.^181^, ^182^ It has been shown that administering NAD^+^ to mice at a specific circadian time (ZT 11) alters the rhythmicity of hepatic mitochondrial biogenesis.^183^ Although direct experimental evidence in dementia remains limited, it is theoretically plausible that circadian oscillations in intracellular NAD^+^ abundance impose a corresponding rhythm on mitochondrial function and ATP output.

When NAD^+^ levels fall during cellular stress, SIRT3 activity declines, leading to hyperacetylation of mitochondrial proteins, reduced electron transport capacity, impaired ATP synthesis, and increased oxidative damage. Circadian control of NAD^+^ bioavailability modulates mitochondrial oxidative function and organismal metabolism via SIRT3.^184^ SIRT3 loss causes widespread mitochondrial protein hyperacetylation, depressed respiration, and decreased basal ATP levels in cells and in vivo. Restoration of SIRT3 activity improves mitochondrial function, lowers ROS, and enhances ATP output. In the Bmal1‐deficient mouse model, SIRT3 activity and mitochondrial oxidative metabolism are impaired, and replenishing NAD^+^ improves mitochondrial function.^184^ In dementia, mitochondrial integrity is compromised, and ATP production is reduced. However, raising NAD^+^ can partially restore mitochondrial performance in animals.^182^, ^185^ Although comprehensive in vivo evidence across dementias from animal models is still emerging, a working model is that age‐related and dementia‐related declines in NAD^+^ weaken SIRT3‐driven mitochondrial ATP production. In contrast, replenishing NAD^+^ could improve ATP generation, support glial energy demand for clearance of pathogenic protein aggregates in disorders such as AD, and help maintain neuronal viability.^179^ As NAD^+^ itself exhibits circadian rhythmicity, SIRT3 activity is also temporally regulated, driving time‐of‐day‐specific changes in acetylation of mitochondrial enzymes, respiratory efficiency, and ATP production.^184^

Numerous lines of evidence indicate that mitochondrial ATP production and SIRT3 activity are disrupted in dementia, and NAD^+^ augmentation can enhance these processes and ameliorate bioenergetic deficits. However, few studies have explicitly examined these effects within a circadian framework in dementia. Because NAD^+^ availability varies across the day and can be experimentally manipulated, the timing of NAD^+^ administration is likely to modulate the phase and amplitude of SIRT3‐dependent deacetylation and, in turn, mitochondrial rhythmicity. Additionally, NAD^+^‐driven changes in SIRT3‐mediated mitochondrial bioenergetics may contribute to broader circadian organization in dementia. Despite limited prior work, these hypotheses are testable and merit systematic investigation.

### 3.5 Neuroinflammation rhythm in dementia

CD38 is a membrane‐associated extracellular enzyme with NAD^+^ glycoside hydrolase and ADP‐ribosyl cyclase activities and is highly expressed in immune cells. It mainly regulates inflammatory signaling, immune responses, and calcium signaling in the brain, with elevated levels observed in neurodegenerative disorders such as AD and PD patients and models.^56^, ^186^, ^187^, ^188^, ^189^, ^190^ CD38 expression also follows a circadian rhythm,^191^ and the loss of the circadian regulator REV‐ERBα/NR1D1 suppresses CD38 transcription, thereby elevating NAD^+^ levels in mouse models.^105^ However, we suspect that when REV‐ERBα/NR1D1 repression weakens, CD38 increases, NAD^+^ oscillations collapse, and sirtuin and PARP‐dependent repair, metabolism, and redox control may deteriorate. Although CD38 inhibition is mechanistically linked to REV‐ERBα/NR1D1, NAD^+^ levels, and inflammation, the current evidence largely emphasizes increases in total NAD^+^ levels and improvements in inflammatory or vascular outcomes. However, existing studies have not directly shown that CD38 modification influences circadian organization within neuroimmune or metabolic systems. Circadian disruption and stressors such as SCN degeneration, hypoxia, and chronic neuroinflammation further suppress NAMPT expression and destabilize the REV‐ERBα/NR1D1‐CD38‐NAD^+^ loop, thereby creating a self‐reinforcing cycle that could amplify neuroimmune signaling, synaptic vulnerability, and cognitive decline (Figure 1). Therapeutically targeting this axis may help restore circadian and metabolic resilience in dementia.

Beyond CD38, NAD^+^ plays a vital role in modulating other inflammation pathways in the brain. With dementia and the progression of aging‐related disorders, neuroimmune homeostasis becomes dysregulated, partly due to the overactivation of the GMP‐AMP synthase stimulator of the interferon gene (cGAS–STING) pathway.^192^, ^193^, ^194^, ^195^ This chronic activation exacerbates inflammation, whereas elevated NAD^+^ suppresses cGAS–STING signaling.^194^, ^196^ In the AD mouse model, NAD^+^ supplementation alleviates neuroinflammation and cellular senescence by inhibiting the cGAS–STING pathway.^194^, ^197^ In tau transgenic mice, cGAS‐STING signaling is activated, and pharmacologic or genetic inhibition of this pathway can restore synaptic integrity and plasticity and improve memory.^198^ NAD^+^‐dependent sirtuins also counteract inflammation: SIRT1 deacetylates nuclear factor kappa‐light‐chain enhancer of activated B‐cell (NF‐κB) p65 subunit, limiting cytokine expression in microglia, while SIRT1 loss triggers neuroinflammation and cognitive decline.^199^, ^200^ In tauopathy models, SIRT1 deficiency contributes to age‐related cognitive decline in neurodegenerative disease, at least in part by epigenetically regulating IL‐1β expression.^199^ Similarly, mitochondrial SIRT3 maintains redox homeostasis and inhibits NLRP3 inflammasome activation. SIRT3 deficiency leads to mitochondrial dysfunction, microglial proliferation, and IL‐1β overproduction, all of which are reversible by SIRT3 activation or NLRP3 inhibition.^201^, ^202^

Furthermore, the neuroimmune system exhibits robust circadian rhythms, which are highly dependent on sleep. In mice, sleep deprivation elevates brain prostaglandin D2 (PGD_2_) levels, promoting its efflux through the ABCC4 transporter and inducing excessive cytokine production that can lead to a cytokine storm‐like response and multi‐organ dysfunction.^203^ Notably, NAD^+^ supplementation has been shown to prevent cognitive decline and microglial activation in mouse models of central sleep apnea by reducing pro‐inflammatory cytokine expression.^204^ Microglia and astrocytes, the brain's primary immune cells, exhibit intrinsic circadian programs that are remodeled in AD, suggesting that disrupted glial rhythms may contribute to disease progression by amplifying inflammation.^21^ However, whether NAD^+^ influences the circadian rhythm of neuroinflammation in dementia or modulates the excessive inflammatory activation associated with sleep disturbances in these conditions remains to be explored. Based on current research, NAD^+^ elevation may represent a promising target for investigation in regulating the immune rhythms of the nervous system.

As mentioned, NAD^+^ is increasingly recognized as a regulator of astrocyte‐ and microglia‐driven neuroinflammation through multiple pathways, and neuroinflammatory processes also show pronounced circadian variation. Accordingly, timed NAD^+^ supplementation may reduce inflammatory activity during the rising phase of the daily cycle, thereby supporting neural homeostasis and neuronal health. In parallel, targeting CD38 expression and rhythmicity may reshape NAD^+^ availability and, in turn, modulate downstream anti‐inflammatory and circadian effects.

## 4 NAD^+^‐TARGETING THERAPIES

### 4.1 NAD^+^ precursor supplementation

**FIGURE 2:**
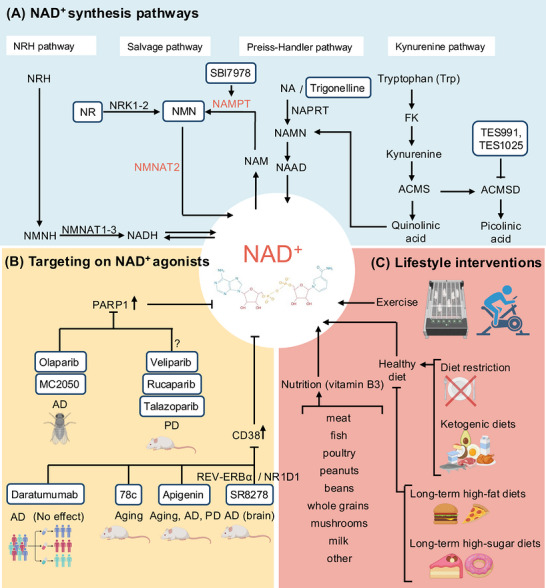
Modulation of NAD^+^ metabolism by synthetic pathways, pharmacologic agents, and lifestyle interventions. (A) The panel depicts the main routes of NAD^+^ synthesis, including the salvage pathways from NR and NAM via NRK1/2, NMNAT2, and NAMPT, the Preiss–Handler pathway from nicotinic acid via NAPRT, and the kynurenine pathway from tryptophan via intermediates such as kynurenine and quinolinic acid. In three main synthetic pathways, NR and NMN, key precursors of NAD^+^, have been widely used in both preclinical and clinical trials. SBI797812 activates NAMPT and increases intracellular NAD^+^. Trigonelline is shown on the nicotinic acid branch as a natural N‐methyl derivative of vitamin B3 that can be converted via NAPRT‐dependent steps into NAD^+^, thereby acting as a precursor in the Preiss–Handler pathway. TES991 and TES1025 are placed beside ACMSD in the tryptophan‐kynurenine pathway because they inhibit ACMSD, diverting ACMS toward quinolinic acid production. (B) The panel summarizes NAD^+^ agonist strategies targeting NAD^+^‐consuming enzymes, including PARP1 inhibitors such as olaparib, MC2050, veliparib, rucaparib, and talazoparib, and their evaluation in AD and PD models. CD38‐targeting approaches such as daratumumab, 78c, apigenin, and the REV‐ERBα modulator SR8278 have been tested in aging and neurodegeneration. (C) The panel illustrates lifestyle interventions that influence NAD^+^, including physical exercise and nutrition that provides vitamin B3 from foods such as meat, fish, poultry, peanuts, beans, whole grains, mushrooms, and milk. Meanwhile, dietary restrictions, including ketogenic diets and chronic high‐fat or high‐sugar diets, can also increase NAD^+^ levels. ACMSD, α‐Amino‐β‐carboxymuconate‐ε‐semialdehyde decarboxylase; NAD^+^, nicotinamide adenine dinucleotide; NAM, nicotinamide; NAMPT, nicotinamide phosphoribosyltransferase; NAPRT, nicotinic acid phosphoribosyltransferase; NR, nicotinamide riboside; PARP1, poly(ADP‐ribose) polymerase 1; REV‐ERBα, reverse c‐erbAα.

Elevating NAD^+^ has been linked to neuroprotective benefits, and it can be achieved by supplying NAD^+^ precursors, limiting NAD^+^ consumption, or enhancing endogenous NAD^+^ biosynthesis. Major NAD^+^ precursors replenish intracellular NAD^+^ pools through distinct biosynthetic routes (Figure 2, Table 2).

**TABLE 2:** Summary of NAD+‐targeting strategies in dementia.

Therapeutic class	Representative strategy	Targeted pathway and mechanism	Key advantages	Major challenges and gaps
NAD^+^ precursor supplementation	NR	Salvage pathway	Consistent safety and target engagement	Variable efficacy, brain penetration unclear, CD38 induction risk
	NMN	Salvage pathway	Consistent safety and target engagement	limited dementia clinical data
	NAM	Salvage pathway	Chemically stable and inexpensive	High doses inhibit sirtuins
	NA	Preiss–Handler pathway	Highly efficient on NAD^+^ supplement	limited neuronal data
NAD^+^ biosynthetic pathway modulators	SBI797812	Activates NAMPT	Targeting capabilities and relieves the feedback inhibition of NAD+	limited neuronal data
	TES991, TES1025	Inhibit ACMSD, diverting ACMS toward quinolinic acid production	Inhibit multiple organs injury	Quinolinic acid neurotoxicity risk
	Trigonelline	Preiss–Handler pathway via NAPRT	Naturally derived, dietary relevance	Limited neuronal data
Indirect NAD^+^‐enhancing compounds	Metformin	AMPK activator, raises NAD^+^ to NADH ratio	Safety profile and broad benefits	Pleiotropy
	AICAR	Activates AMPK and mitochondrial biogenesis	Strong metabolic effects	Pleiotropy
	Resveratrol	Increases mitochondrial NAD^+^ to NADH ratio	Safety profile and broad benefits	Pleiotropy
	P7C3‐A20	Restore brain NAD^+^ homeostasis	Target to AD and improve AD model cognition	In vitro toxicity
NAD^+^‐consuming enzyme inhibition	Olaparib	PARP1 inhibitor	Reduced Aβ_4__2_ aggregation and exhibited neuroprotective effects in AD model	Disrupt DNA damage response pathways; long‐term safety detection and existing preclinical studies remain limited
	Veliparib	PARP1 inhibitor	Decrease α‐synuclein aggregation and neuronal toxicity in PD model	
	Rucaparib	PARP1 inhibitor		
	Daratumumab	CD38 inhibitor	In clinical trial directly assessing CD38‐targeted therapy in AD	Limited nervous system targeting and may suppress immune function and impair social behavior
	78c	CD38 inhibitor	Ameliorated blood–brain barrier disruption and cognitive decline in mice	
	Apigenin	CD38 inhibitor	In PD, reduces oxidative stress signatures and improves locomotor performance	
	SR8278	REV‐ERB antagonist and suppresses CD38 in astrocytes	Reduces tau pathology in mice	Complex effect on circadian rhythm and negative effect on other organs
Lifestyle and chronobiological interventions	Endurance exercise	Activates AMPK and PGC1α, and increases NAMPT	Elevates AMP and calcium ion concentrations, activates AMPK and PGC1α	Not user‐friendly for older adults, dementia patients, or individuals with mobility limitations
	Dietary restriction	Elevates NAD^+^ to NADH ratio	Activate SIRT1 in different organs and activate AMPK	Difficult to imply on patients with advanced dementia; would require long‐duration clinical trials in older populations
	Vitamin B3‐rich foods	Salvage pathway and Preiss–Handler pathway	Safe, effective in everyday life, and without side‐effects	Providing modest amounts of dietary NAD plus intermediates at very low doses

Abbreviations: ACMS, α‐amino‐β‐carboxymuconate‐ε‐semialdehyde; ACMSD, α‐amino‐β‐carboxymuconate‐ε‐semialdehyde decarboxylase; AICAR, 5‐aminoimidazole‐4‐carboxamide ribonucleotide; AMP, adenosine monophosphate; AMPK, AMP‐activated protein kinase; CD38, cluster of differentiation 38; NA, nicotinic acid; NADH, nicotinamide adenine dinucleotide in reduced form; NAM, nicotinamide; NAMPT, nicotinamide phosphoribosyltransferase; NAPRT, nicotinic acid phosphoribosyltransferase; NMN, nicotinamide mononucleotide; NR, nicotinamide riboside; PARP1, poly ADP ribose polymerase 1; PGC1α, peroxisome proliferator activated receptor γ coactivator 1α.

Clinical trials of NAD^+^ precursors show consistent safety and target engagement but variable efficacy signals. In older adults with MCI, administration of nicotinamide riboside (NR) for 10 weeks induced circulating NAD^+^ metabolites without improving cognition,^205^, ^206^ and in early AD, 48 weeks of exposure to high‐dose nicotinamide was safe but did not shift core biomarkers.^207^ A phase II trial of a multi‐ingredient metabolic activator mix that included NR reported cognitive and multi‐omics improvements over 84 days. Still, the specific contribution of NAD^+^ elevation could not be isolated,^208^ while ongoing clinical studies are testing NR and NMN in MCI and mild AD. In PD, short clinical trials show central and peripheral engagement with increased brain NAD^+^ by phosphorus magnetic resonance spectroscopy and about fivefold higher blood NAD^+^. However, slight clinical improvement was observed only in patients who showed a robust increase in brain NAD^+^, and the overall clinical signals remained modest and may have been confounded by the timing of levodopa.^209^, ^210^ Overall, current evidence supports the pharmacologic activity of NAD^+^ boosting in humans, while definitive neuroprotective efficacy still requires larger and longer trials. Despite the importance of NR supplementation, preclinical research found that improper NR supplementation led to CD38 induction, decreasing NAD^+^ levels and causing unexpected physiological disadvantages under the apoE knockout condition.^211^ It implies that administering NR supplements to raise endogenous NAD^+^ levels should be done carefully to treat NAD^+^‐related disorders. Definitive evidence of durable clinical benefit across neurological diseases will require larger and longer studies.

Human studies connecting NAD^+^ prodrugs to sleep and circadian outcomes are slowly increasing in number. In chronopharmacology, a randomized trial reported that NMN treatment in the afternoon for 12 weeks improved sleep quality, reduced fatigue, and enhanced physical function in older adults compared with placebo.^212^ A registered trial is evaluating oral NMN for chronic primary insomnia with validated sleep scales and safety endpoints.^213^ Two NR studies are also under way in older adults and healthy volunteers that directly target sleep outcomes and should clarify whether raising NAD^+^ improves objective and subjective sleep quality. A clinical trial in older adults reports positive effects on aging‐induced sleep, diurnal activity, and motor function.^212^, ^214^ Ongoing NAD^+^ precursor‐related trials will be important to establish durability and clinical relevance.

Clinical studies suggest that NAD^+^ precursors may help regulate circadian rhythms, but findings regarding dementia‐related cognition and neuropathology remain mixed. Because dementia is multifactorial and heterogeneous, circadian disruption can act as both a contributing factor and a clinical manifestation of disease progression. Accordingly, NAD‐based interventions may yield greater benefit in patients with well‐characterized circadian abnormalities, particularly when cognitive impairment is also present. Future studies should characterize sleep and circadian phenotypes at enrollment, measure circadian‐related biomarkers, and then perform stratified analyses by circadian profile to better identify likely responders and define efficacy patterns. One likely reason is reduced exposure in humans, as trials often use smaller doses and shorter treatment periods than rodent studies, which may yield weaker biological effects. Second, in a clinical trial of newly diagnosed PD, daily oral NR at 1000 mg for 30 days increased brain NAD^+^ levels as assessed by phosphorus magnetic resonance spectroscopy, yet slight clinical improvement was observed only among participants who exhibited a robust increase in brain NAD^+^.^210^ This suggests that the insufficient elevation of brain NAD^+^ could limit downstream cognitive benefits. Optimizing formulations and delivery strategies to enhance neural exposure and target engagement may therefore be essential to improve therapeutic efficacy. Additionally, the therapeutic window differs: Animal studies often initiate high‐dose supplementation before pathology develops, whereas clinical trials typically begin after diagnosis, when established disease may limit the impact of NAD^+^ augmentation on symptoms or pathology. Finally, chronopharmacology is an important consideration because NAD^+^ metabolism and the circadian system interact bidirectionally. As a result, administration at different circadian phases may produce distinct therapeutic effects, particularly for outcomes related to daily activity and sleep‐wake regulation. Animal studies suggest that NAD^+^ supplementation during the active phase improves metabolic function, whereas administration during the rest phase may attenuate these benefits or even disrupt metabolic rhythmicity.^183^ Clinical evidence further indicates that NMN administration in the evening, particularly after 6:00 p.m., may be more effective in improving drowsiness and sleep quality.^212^ Although NAD^+^ precursors have been widely studied, systematic investigation of the optimal timing of administration remains limited.

Currently, no recorded or registered trials are evaluating NAD^+^‐related interventions with sleep or circadian outcomes in AD, PD, or other dementias, because most studies to date have emphasized safety, pharmacokinetics, or global cognition rather than sleep. Given that the clinical efficacy of NR and NMN in dementia remains unproven, investigators have prioritized small mechanistic studies and sleep research in healthy individuals or older adults before launching disease‐specific trials focused on sleep and circadian rhythms.

### 4.2 The new prodrugs on NAD^+^ biosynthetic pathway

Oral or injectable NAD^+^ precursors moderately elevate tissue NAD^+^, as circulatory degradation of NAD^+^ and hepatic first‐pass metabolism reduce the functional precursor available for NAD^+^ synthesis in target tissues.^215^, ^216^ Beyond common nutritional and vitamin B3‐derived precursors, small‐molecule drugs that act within the NAD^+^ biosynthetic network have recently been identified (Figure 2, Table 2). For example, the NAMPT activator SBI797812 binds NAMPT and redirects its activity toward NMN production while relieving NAD^+^ feedback inhibition to elevate intracellular NAD^+^ levels in cultured cells and in mouse liver in vivo.^217^ In parallel, inhibitors of α‐Amino‐β‐carboxymuconate‐ε‐semialdehyde decarboxylase (ACMSD), an enzyme at a branch point of the kynurenine pathway, represents an emerging pharmacological strategy. Compounds such as TES991 and TES1025 divert tryptophan flux toward quinolinic acid and de novo NAD^+^ synthesis, thereby increasing NAD^+^ levels in liver and kidney, improving mitochondrial gene expression, and protecting mice from fatty liver and cisplatin‐induced kidney injury.^218^, ^219^ In addition, naturally derived components such as the coffee alkaloid trigonelline are metabolized through the Preiss–Handler pathway via phosphoribosyltransferase (NAPRT) and promote NAD^+^ biosynthesis through both direct and indirect mechanisms while enhancing muscle NAD^+^ content and function in aged mice.^220^

Some compounds that do not directly target NAD^+^ enzymes can still raise NAD^+^ availability or the NAD^+^‐to‐NADH ratio by improving energy sensing and mitochondrial function. For example, AMPK activators such as metformin and AICAR increase the ratio of NAD^+^ to NADH in skeletal muscle and other tissues in mice.^221^ Resveratrol can stimulate mitochondrial complex I, elevate the mitochondrial ratio of NAD^+^ to NADH, and enhance SIRT3‐dependent oxidative metabolism.^222^ The recently identified neuroprotective compound P7C3‐A20 ameliorated pathology in a 5xFAD mouse model, potentially by restoring brain NAD^+^ homeostasis.^223^ These indirect strategies highlight how modulating energy sensors and mitochondrial dynamics can effectively enhance NAD^+^ bioavailability without directly targeting NAD^+^ biosynthetic enzymes.

A major limitation of current potential new prodrugs, based on the NAD^+^‐boosting strategies, is that most studies document increased NAD^+^ primarily in peripheral tissues, while direct quantitative evidence for elevated NAD^+^ within the central nervous system remains limited (especially on how NAD^+^ is changed in different brain regions and in different brain cell types). Accordingly, it is unclear whether new candidate prodrugs cross the blood–brain barrier at concentrations sufficient to meaningfully augment brain NAD^+^ pools. Currently, published pharmacokinetic profiles frequently emphasize plasma or hepatic exposure and rarely include measurements in brain tissue, cerebrospinal fluid, or region‐specific samples. Therefore, applying new NAD^+^ boosting prodrugs in dementia will require additional experimental studies that directly and quantitatively demonstrate central target engagement.

### 4.3 Targeting on NAD^+^‐consuming enzymes, PARP1, and CD38

Inhibiting key NAD^+^‐consuming enzymes can elevate intracellular NAD^+^ levels and shift cellular metabolism toward a sirtuin‐dependent program, with PARP1 and CD38 being the most extensively studied targets (Figure 2, Table 2). Numerous studies have demonstrated that suppressing PARP1 activity decreases nuclear NAD^+^ consumption, thereby enhancing NAD^+^ and SIRT1 activity while promoting mitochondrial biogenesis and respiration in cellular and animal models.^54^, ^160^, ^224^ Currently, PARP inhibitors, such as olaparib, niraparib, rucaparib, talazoparib, and veliparib, are primarily applied in oncology.^225^, ^226^, ^227^ In breast cancer mice, olaparib has been reported to modulate circadian rhythms while suppressing tumor progression.^228^ However, preclinical study has demonstrated the neuroprotective potential of PARP1 inhibitors in neurological disorders. For instance, the PARP1 inhibitors olaparib and MC2050 reduced Aβ_42_ aggregation and exhibited neuroprotective effects in the AD Drosophila model.^229^ In PD models, veliparib, rucaparib, and talazoparib were shown to decrease α‐synuclein aggregation and neuronal toxicity in cultured neurons.^151^ Additionally, multiple PARP1 inhibitors have been reported to attenuate neuroinflammation and protect neurons from ischemic injury in mouse models of stroke.^230^, ^231^ Likewise, the anti‐inflammatory actions of PARP1 inhibitors may therefore hold promise for treating dementia.^232^ Beyond the central nervous system, olaparib has also been shown to protect retinal ganglion cells from chronic intraocular pressure in vivo and to alleviate hypoxia‐induced mitochondrial‐associated membrane disruption and mitochondrial dysfunction in vitro.^233^

No PARP1 inhibitors are currently in clinical development for dementia or brain aging, largely owing to safety concerns, the availability of alternative therapeutic strategies, and insufficient preclinical validation. First, the essential role of PARP1 in DNA repair means its inhibition can disrupt DNA damage response pathways, leading to adverse effects such as anemia, thrombocytopenia, neutropenia, nausea, and fatigue in clinic.^234^, ^235^ Although PARP1 inhibitors can cause adverse effects, they have produced promising outcomes in animal models of dementia. When positioned as a short‐term, low‐dose intervention for acute events linked to PARP1 overactivation and NAD^+^ depletion, such as early oxidative stress or ischemic injury, rather than as a long‐term therapy, these agents may offer an acceptable therapy. In addition, improving nervous system targeting may further reduce systemic exposure and thereby mitigate side effects. Second, as an alternative, research on dementia has favored milder NAD^+^‐enhancing strategies, such as nicotinamide and NMN supplementation. Third, existing preclinical studies remain limited. While animal experiments suggest neuroprotective effects through reduced NAD^+^ depletion, these findings are short‐term and based on artificial models, providing insufficient evidence for long‐term efficacy and safety in humans.

CD38 inhibitors are under active clinical investigation for various diseases, primarily as monoclonal antibodies that either deplete CD38‐expressing cells or block CD38 enzymatic activity. Among these, daratumumab and isatuximab have been approved for the treatment of multiple myeloma, while several other candidates are being evaluated for the treatment of autoimmune disorders and for transplant‐related applications.^236^, ^237^ To date, the only clinical trial directly assessing CD38‐targeted therapy in AD is a small, open‐label study using subcutaneous daratumumab.^238^ This trial demonstrated target engagement in peripheral T cells and an acceptable safety profile but did not show any improvement in cognitive function.^238^ From a pharmacological perspective, multiple preclinical studies have demonstrated that inhibiting CD38 enzymatic activity preserves brain NAD^+^ levels, mitigates neuroinflammation, protects neurovascular units, and improves behavioral outcomes in neuroprotection.^45^, ^59^, ^239^ The small‐molecule 78c, a selective CD38 inhibitor in the neurovascular system, restores NAD^+^ by inhibiting CD38 with 78c ameliorated blood–brain barrier disruption and cognitive decline in mice.^240^ Preclinical studies suggest that small‐molecule CD38 inhibitors, including apigenin and quercetin, can increase cellular NAD^+^ levels and produce neuroprotective or anti‐inflammatory effects.^241^, ^242^, ^243^, ^244^ Clinical studies have shown that high‐dose apigenin may improve sleep in patients with chronic primary insomnia.^245^ In *Drosophila* expressing human α‐synuclein, apigenin reduces oxidative stress signatures and improves locomotor performance.^246^ Rodent studies further report sedative‐like behavioral effects, improved learning and memory in aged mice, and suppression of neuroinflammatory brain gene programs.^247^ These findings, together with evidence that apigenin elevates tissue NAD^+^ consistent with CD38 inhibition, support its potential to modulate metabolic and immune pathways implicated in age‐related sleep disruption and cognitive decline. In a vascular dementia model, targeting CD38 in brain endothelial cells alleviated cerebral hypoperfusion‐induced reductions in blood flow, increased barrier permeability, and memory impairment.^240^ In a neuroinflammation model, pharmacological CD38 inhibition by apigenin‐elevated NAD^+^ levels, reduced cytokine production in microglia and astrocytes following lipopolysaccharide stimulation, and attenuated glial activation, thereby slowing neurodegenerative processes.^244^ A recent study demonstrated that inhibiting circadian repressor REV‐ERBα/NR1D1, either through genetic deletion or with the REV‐ERBs antagonist drug SR8278, reduced tau pathology in mice and suppressed CD38, thereby elevating NAD^+^ levels,^105^ and underscored its therapeutic potential.^45^

CD38 inhibitors have not yet advanced rapidly into clinical trials for dementia or brain aging, mainly due to challenges related to delivery, safety, and translational feasibility. The first major obstacle is targeted delivery strategies, which limit drug penetration into the central nervous system. For example, the CD38‐targeting antibody daratumumab exhibits minimal brain exposure in both humans and animal models, with case reports showing cerebrospinal fluid concentrations below therapeutic levels.^248^ A second concern is safety: CD38 inhibitors can suppress immune function and social behavior, risks that are particularly concerning in elderly and dementia populations lacking clear therapeutic benefit.^236^, ^249^ Third, although preclinical studies have linked CD38 upregulation to neuroinflammation, its translation into clinical practice has been slow, due to the limited maturity of biomarkers reflecting central CD38 activity. Despite the encouraging findings from preclinical research, further validation and improved strategies for brain targeting are essential before CD38 inhibitors can be safely and effectively applied in neurodegenerative disease therapy. However, alternative CD38‐targeting agents with more favorable safety profiles have also been reported, including apigenin, as noted above.

Currently, although mechanistic studies indicate that PARP1 and CD38 inhibitors can modulate NAD^+^ homeostasis, neuroinflammation, and neuronal stress responses, evidence that they improve circadian disruption or sleep abnormalities remains limited. These agents therefore remain at an exploratory stage, and studies that prioritize circadian and sleep endpoints will be necessary to define their therapeutic potential.

### 4.4 Lifestyle interventions to boost NAD^+^

Approaches to increasing NAD^+^ levels include not only pharmacological interventions but also behavioral approaches such as circadian regulation, physical exercise, and fasting. Endurance training upregulates NAMPT, thereby enhancing muscle repair. In both humans and mice, repeated training can approximately double skeletal muscle NAMPT abundance and augment the activity of SIRT signaling pathways that depend on NAD^+^ (Figure 2, Table 2).^250^, ^251^, ^252^, ^253^ Mechanistically, muscle contraction elevates AMP and calcium ion concentrations, activates AMPK and PGC1α, and consequently increases NAMPT.^251^ The timing of exercise further modulates circadian oscillations in muscle metabolic pathways and whole‐body energy homeostasis.^254^ As mentioned, evidence from animal models and human studies indicates that regular exercise functions as a rhythmic metabolic stressor that promotes NAD^+^ production, potentially reactivates SIRT1‐dependent circadian clock control, and partly restores the daily temporal organization of NAD^+^ signaling.

Dietary restriction, a cornerstone intervention for promoting metabolic health, fundamentally reshapes NAD^+^ redox balance and activation across species. In rodents, ketogenic diets induce hippocampal NAD^+^/NADH ratios within several days of ketosis onset and sustain them during prolonged treatment, enhancing neuronal resilience.^255^, ^256^, ^257^ Similarly, calorie restriction boosts cellular NAD^+^/NADH ratios, activating Sir2 (the ortholog of SIRT1 in yeast)^258^ and mammalian SIRT1 in brain, adipose, liver, and kidney to regulate cell survival pathways.^259^ Fasting further engages energy‐sensing pathways by activating AMPK, which phosphorylates clock components such as casein kinase I and CRY to promote PER2 stability and circadian timing,^260^ while the molecular circadian system reciprocally drives rhythmic NAMPT expression and NAD^+^ oscillations.^176^ Collectively, strategic timing of caloric intake thus optimizes NAD^+^ rhythms and bioavailability for anti‐aging benefit. Dietary patterns influence systemic NAD^+^ availability through multiple metabolic pathways. Nutritionally, NAD^+^ homeostasis is shaped primarily by intake of vitamin B3, tryptophan‐rich protein sources, small amounts of newly described food precursors, and overall diet quality. NAD^+^ is synthesized from NA and NAM through the Preiss–Handler pathway and the salvage pathway respectively, with vitamin B3‐rich foods, including meat, fish, poultry, peanuts, beans, whole grains, mushrooms, milk, and other dairy products, constituting primary nutritional sources for endogenous NAD^+^ biosynthesis.^261^ Trace amounts of NR and NMN have also been detected in foods such as meat, vegetables, milk, and yeast‐containing products, providing modest dietary NAD^+^ intermediates that remain far below pharmacological supplementation doses.^256^, ^257^ In addition, dietary patterns also regulate NAD^+^ turnover, as we previously discussed. Long‐term high‐fat or high‐sugar diets and obesity can activate NAD^+^‐consuming enzymes, including PARP1 and CD38. In contrast, energy restriction and weight loss increase the NAD^+^/NADH ratios and enhance NAD^+^‐dependent signaling in metabolic tissues.^262^, ^263^ Taken together, these data indicate that nutritional strategies to support NAD^+^ should combine adequate vitamin B3 and tryptophan intake from diverse whole food sources with carefully planned dietary patterns that promote ketone body metabolism when appropriate and limit highly processed, sugar‐rich, or fat‐rich foods that chronically increase NAD^+^ consumption.

### 4.5 Future translational research directions

Despite substantial advances in NAD^+^ supplementation strategies and the lack of major adverse effects reported in human trials, these approaches have not yet translated into meaningful neurological therapies for dementia. Nonetheless, current clinical research highlights several priorities for future work. First, several NAD^+^‐elevating agents have been shown to increase peripheral NAD^+^ levels, for example, in blood and muscle, yet their ability to raise NAD^+^ in the brain remains uncertain.^264^, ^265^, ^266^ Consequently, the effects of the agents on the nervous system and dementia require further investigation. Second, optimal dose, administration timing and duration, and context dependence remain uncertain. To better approximate conditions used in animal studies, NAD^+^ precursor therapies may require earlier initiation, longer treatment durations, and higher doses to determine whether comparable therapeutic benefits can be achieved in humans with dementia. Meanwhile, the therapeutic application of PARP1 inhibitors warrants further investigation into the dose and timing of administration, as PARP1 is involved in other biological processes. Their efficacy and adverse effect profiles may differ across clinical contexts. Third, improvements in NAD^+^ availability, SIRT1 activity, and metabolic health often co‐occur with circadian realignment, implying the presence of multiple feedback loops between metabolic state and clock regulation. However, much of the supporting evidence comes from animal models or peripheral tissues, and evidence for NAD^+^ rhythmicity in humans remains limited. Fourth, one major challenge in elucidating NAD^+^‐related mechanisms and translating NAD‐based interventions to humans is the absence of accurate and accessible methods for NAD^+^ detection and labeling in vivo, specifically in the brain. Although functional magnetic resonance imaging can be used for monitoring, it remains difficult to achieve long‐term continuous assessment across the circadian cycle. Thus, the development of reliable biomarkers that faithfully reflect brain NAD^+^ metabolism, NAD^+^‐dependent enzyme activity, and circadian NAD^+^ dynamics is essential for advancing NAD^+^ research and enabling effective clinical translation.

Finally, future research should further delineate how NAD^+^‐enhancing interventions influence neurological function and dementia‐related pathology. Trial designs should integrate circadian biology with lifestyle factors and use rigorously designed randomized controlled trials of sufficient duration. Efficacy assessment should incorporate sleep and circadian‐sensitive endpoints, including actigraphy‐derived measures, polysomnography metrics, and circadian phase markers. A major challenge is that dementia‐related pathological processes can also vary across the circadian cycle, complicating clinical assessment. Imaging is poorly suited for long‐term, high‐frequency tracking, whereas repeated cerebrospinal fluid collection is invasive. Although these approaches are not yet in routine clinical use, emerging clinical and preclinical evidence supports an association between peripheral blood biomarkers and dementia‐related pathology. Because blood sampling is minimally invasive and repeatable, longitudinal monitoring with highly sensitive blood biomarkers may provide a practical approach to capturing circadian variation in dementia‐related pathological dynamics.

## 5 CONCLUSIONS AND CHALLENGES

As populations age, dementia is becoming increasingly common and imposes major healthcare and socioeconomic burdens.^267^, ^268^ Beyond directly targeting Aβ and tau, growing efforts are exploring NAD^+^ augmentation to modulate circadian rhythms, metabolism, and energy homeostasis.^45^, ^223^, ^269^, ^270^

Advances in circadian biology are shifting the management of sleep and circadian disturbances in dementia from symptomatic control toward mechanistic modulation of the endogenous clock. Morning light exposure and appropriately timed melatonin are often considered first‐line options in older adults due to favorable safety profiles and the cognitive and dependency risks of many sedative agents, with sedative hypnotics such as clonazepam generally reserved for refractory cases.^271^, ^272^, ^273^ In parallel, precision approaches are advancing through polysomnography‐based physiological biomarkers such as REM muscle atonia, electromyographic features, and spectral EEG signatures, which, when integrated with molecular and imaging biomarkers such as cerebrospinal fluid α‐synuclein and metabolic profiling, improve early diagnosis and risk stratification for synucleinopathies, and inform disease‐modifying strategies targeting NAD^+^ metabolism, sirtuin signaling, core clock gene pathways, and neuroinflammatory cascades. Overall, optimizing sleep and circadian dysfunction management in dementia improves sleep quality, behavioral symptoms, and caregiver burden and may slow cognitive decline, supporting its central role in integrated dementia care.

This review is the first to integrate dementia subtype‐specific circadian rhythm phenotypes with NAD‐dependent pathways and to examine the potential mechanisms through which NAD^+^ supplementation may influence disease progression by correcting circadian disturbances. By synthesizing the current evidence and identifying its limitations, this review also seeks to clarify priorities for future mechanistic studies and clinical translation. In dementia, this reduction reflects both impaired NAD^+^ synthesis, including compromised NAMPT‐dependent salvage pathways, and increased NAD^+^ consumption by enzymes such as PARP1 and CD38. Together, these changes diminish SIRT1 and SIRT3 activities, thereby disrupting circadian transcriptional programs. Although NAD^+^‐boosting strategies have shown context‐dependent benefits in dementia, their impact on circadian regulation remains insufficiently defined and requires further systematic investigation.^22^

## CONFLICT OF INTEREST STATEMENT

Evandro Fei Fang is a co‐owner of Fang‐S Consultation AS (Organization No.: 931 410 717) and NO‐Age AS (Organization No.: 933 219 127); he has a material transfer agreement (MTA) with LMITO Therapeutics lnc. (South Korea), a Cooperative Research and Development Agreement arrangement with ChrgmaDex (USA), a commercialization agreement with Molecule AGNITADAO, a MTA with GeneHarkgr (Hong Kong) Biotechnologies Limited, and a data license option agreement with Hong Kong Longevity Science Laboratory (Hong Kong); he is a consultant to MindRank Al (China), NYO3 (Norway), AgeLab (Vitality Nordic AS, Norway), and Hong Kong Longevity Science Laboratory (Hong Kong). Harald Hrubos‐Strøm was partially supported by Lilly and Novo Nordisk. Other co‐authors have nothing to declare. Author disclosures are available in the Supporting Information.

## Supporting information



Supporting Information
